# Supplementary material for: Combined Treatment with Evogliptin and Temozolomide Alters miRNA Expression but Shows Limited Additive Effect on Glioma
Source: Int J Mol Sci. 2025 Sep 28;26(19):9508. doi: 10.3390/ijms26199508 (PMC12525080; doi:10.3390/ijms26199508)
Supplement: Supplementary file 1 [file ijms-26-09508-s001.zip › ijms-3821190-supplementary.pdf]

U87

U87
Control vs. TMZ

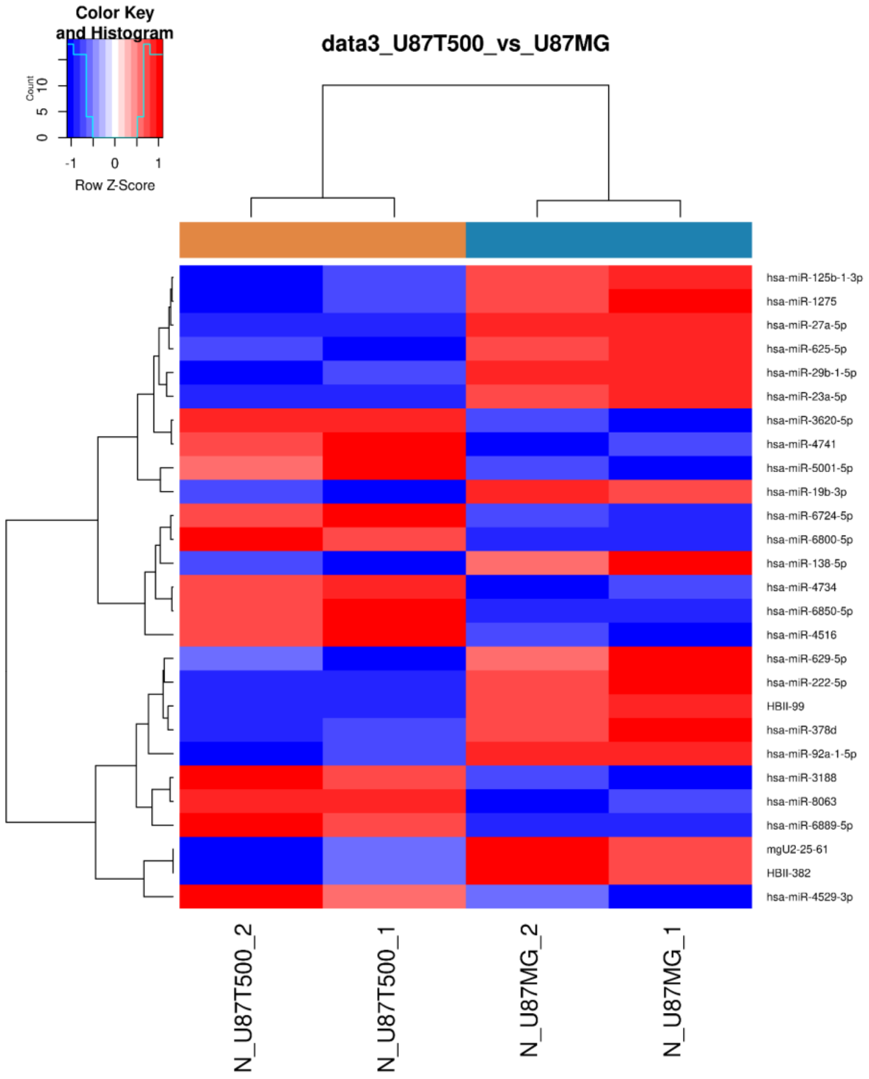

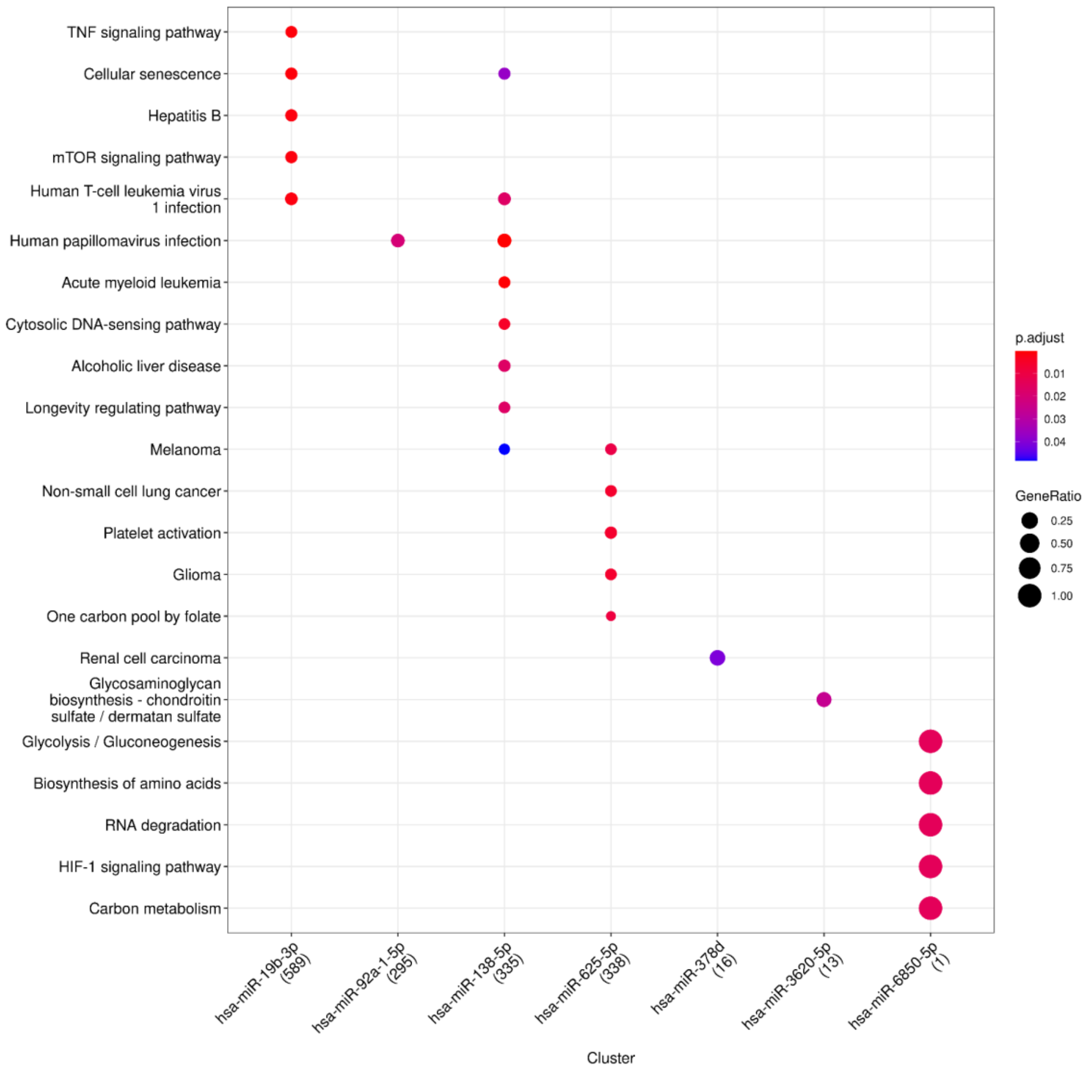

U87
Control vs. Evo

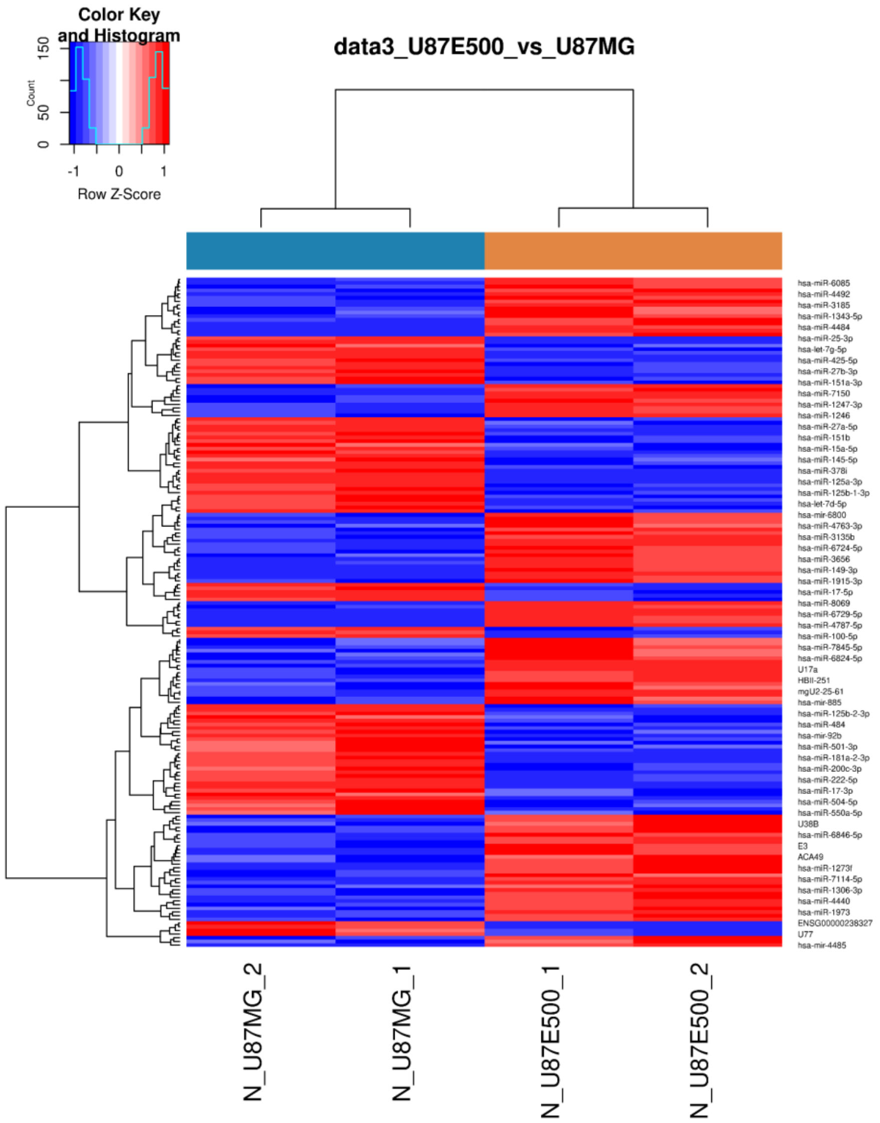

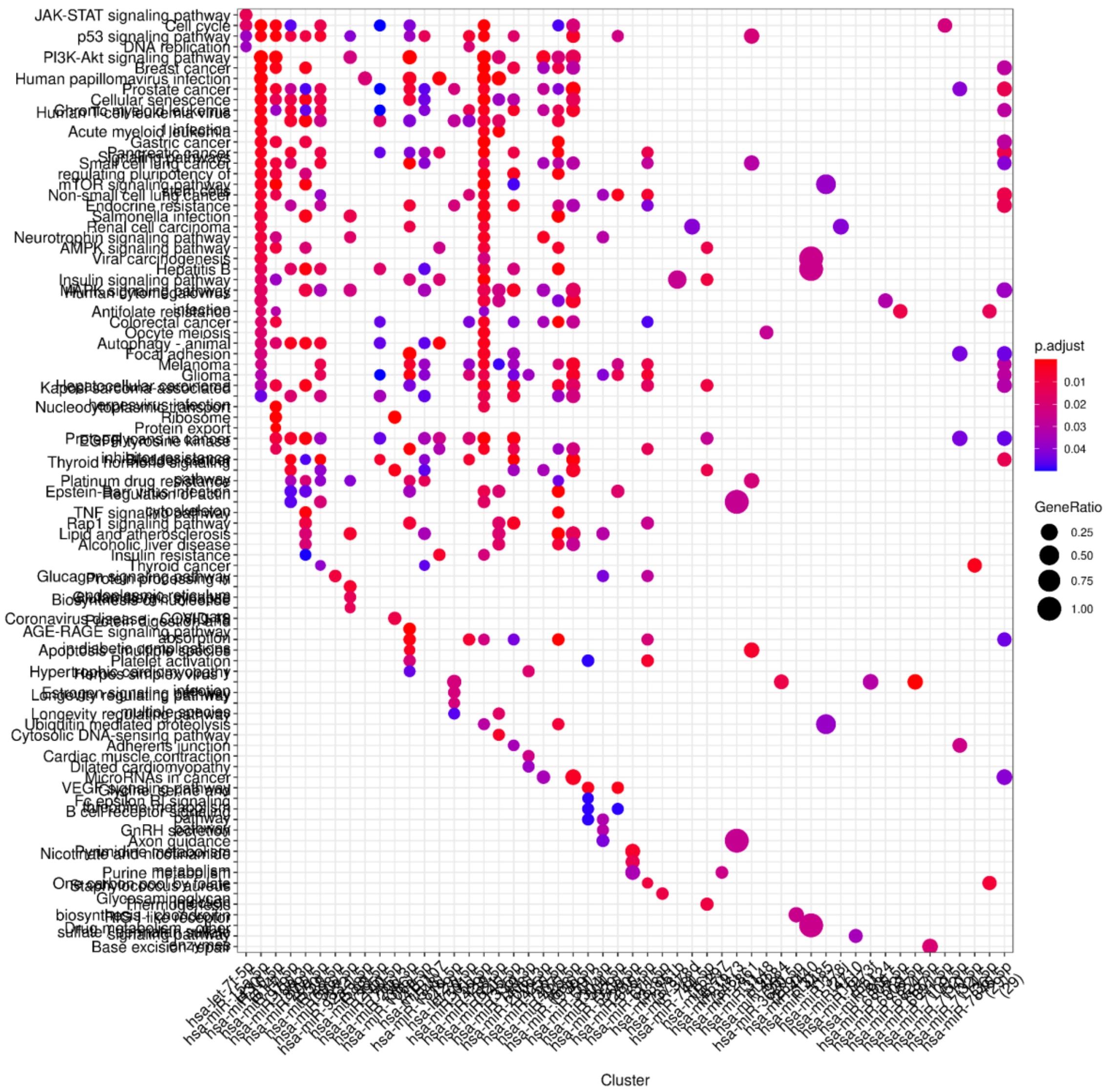

U87
Evo vs. TMZ

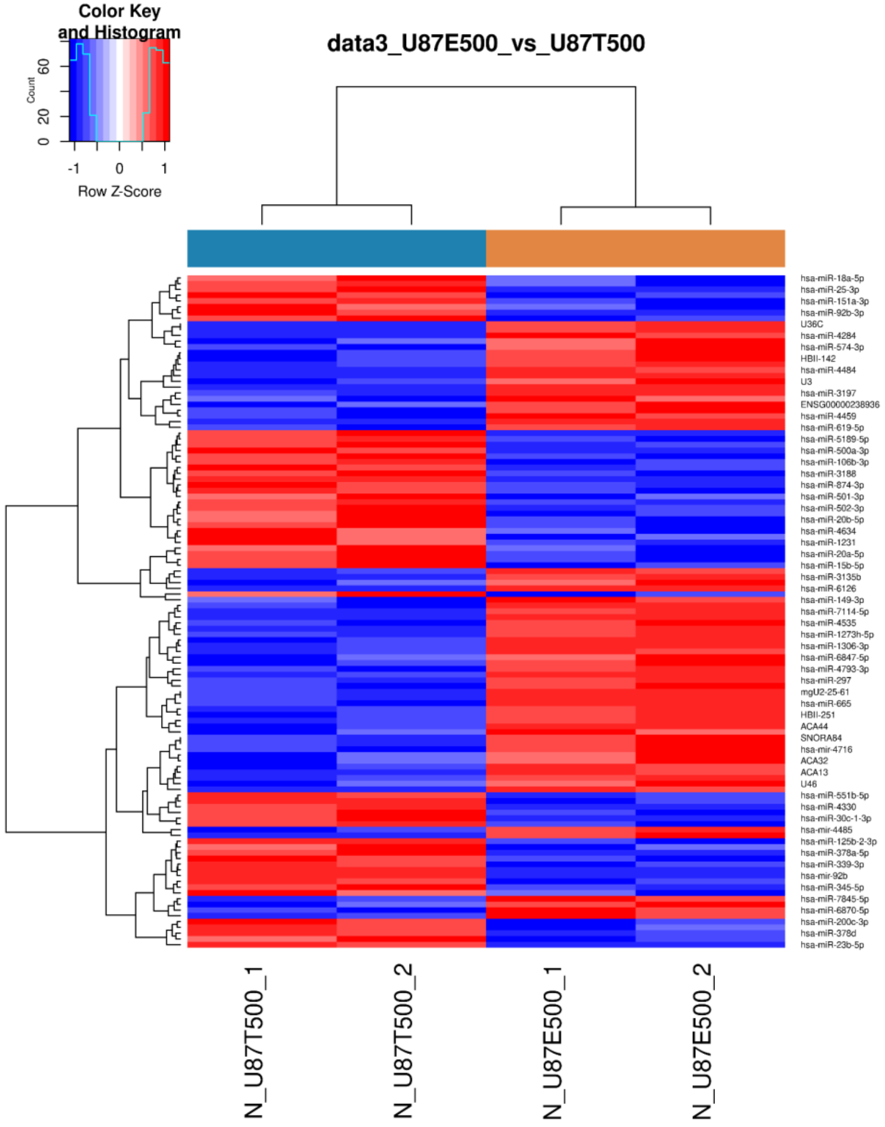

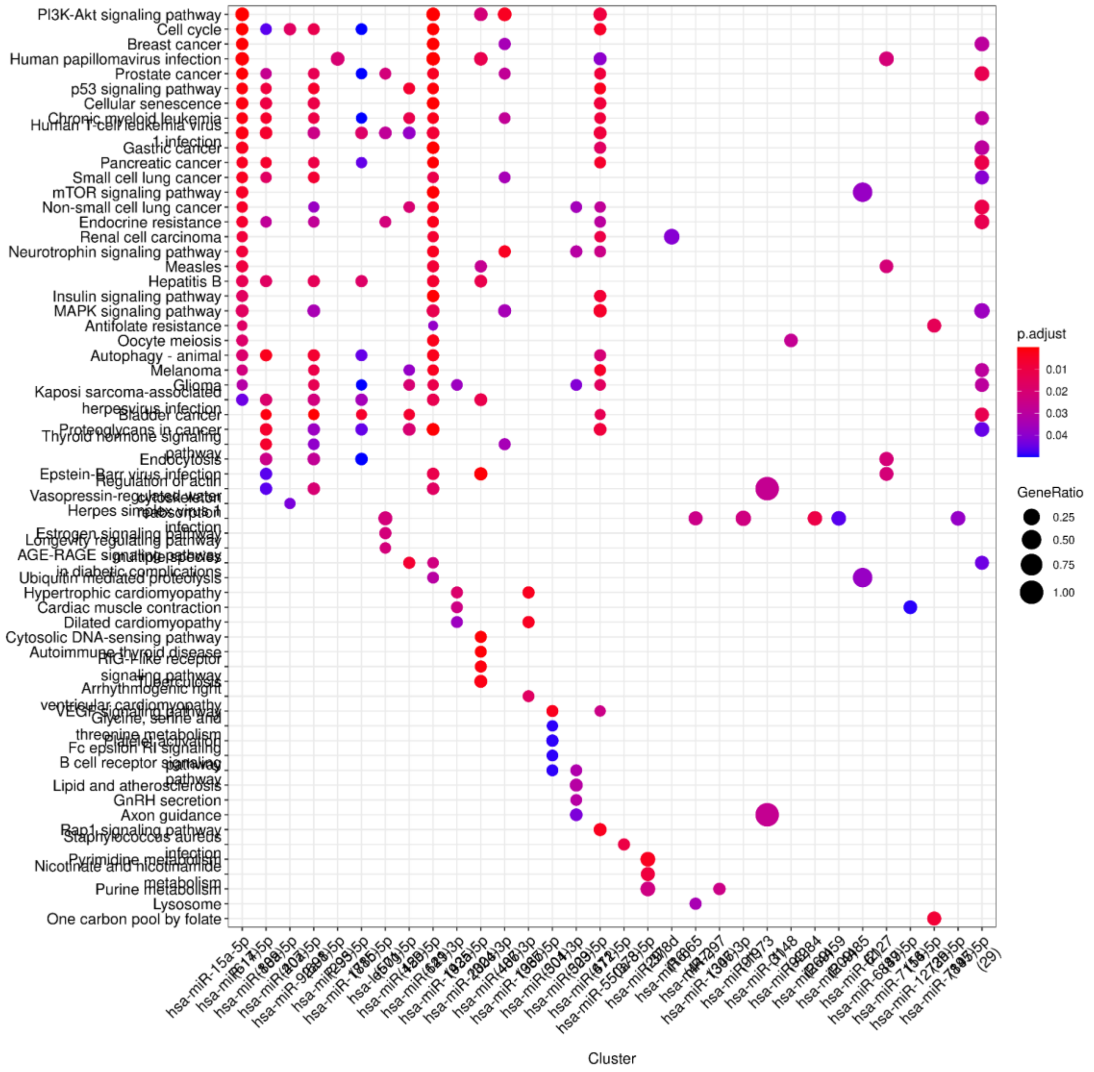

U87
Evo vs. Evo/TMZ

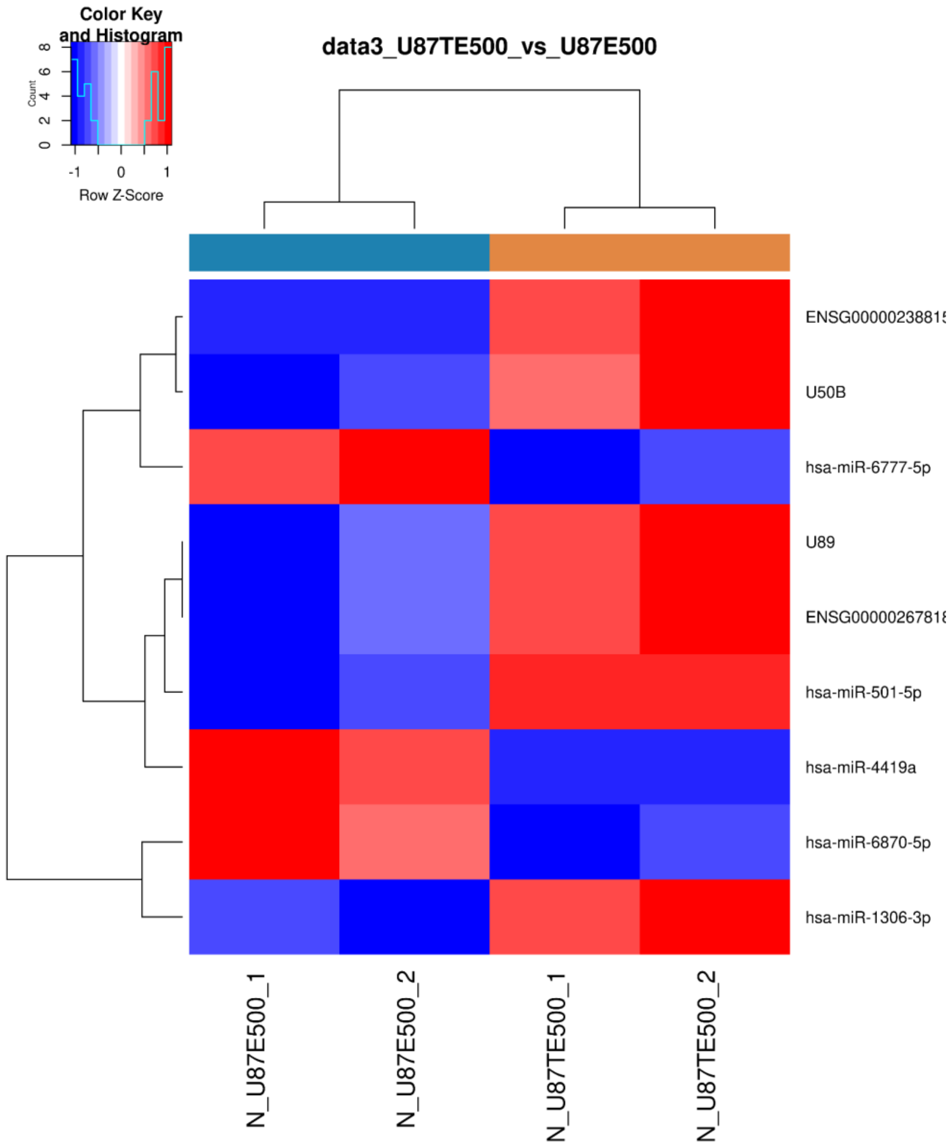

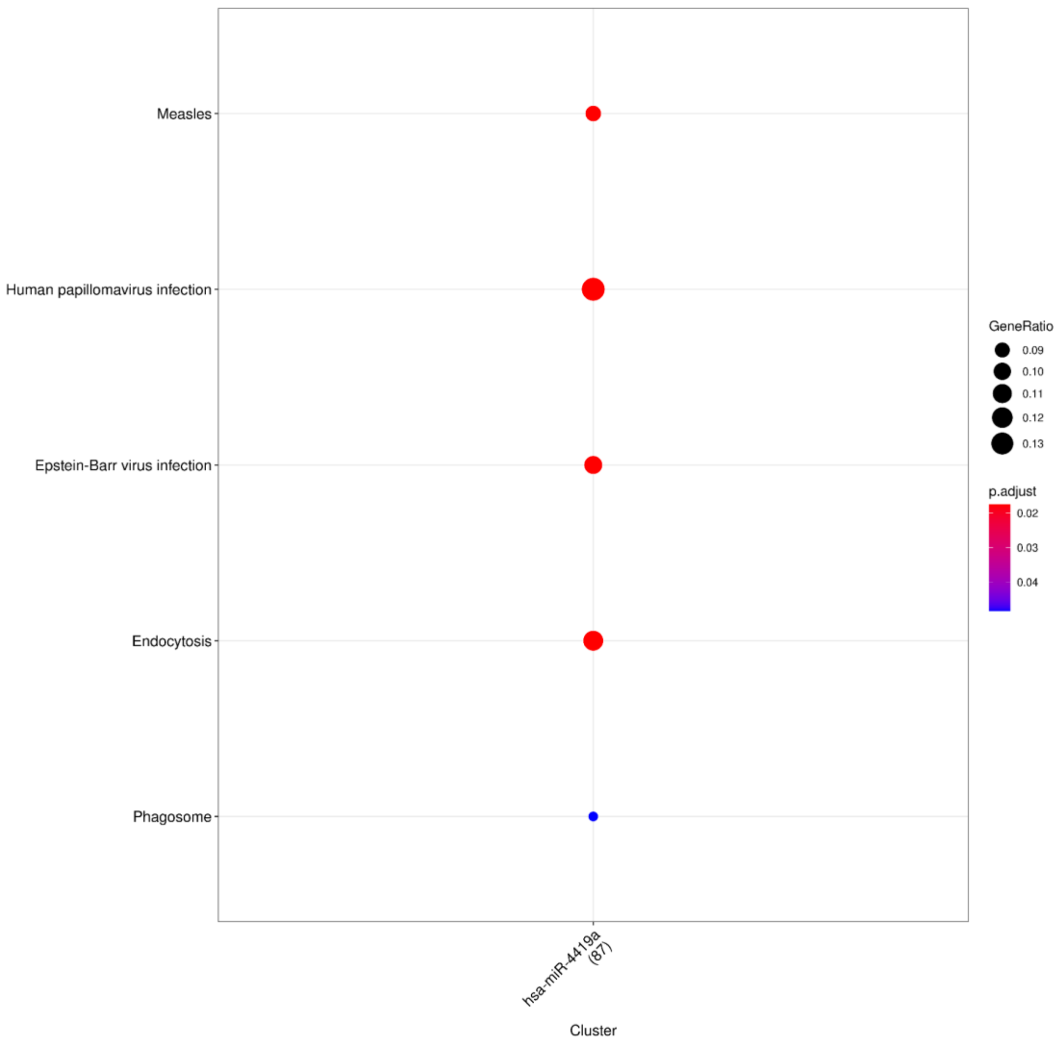

U87
Control vs. Evo/TMZ

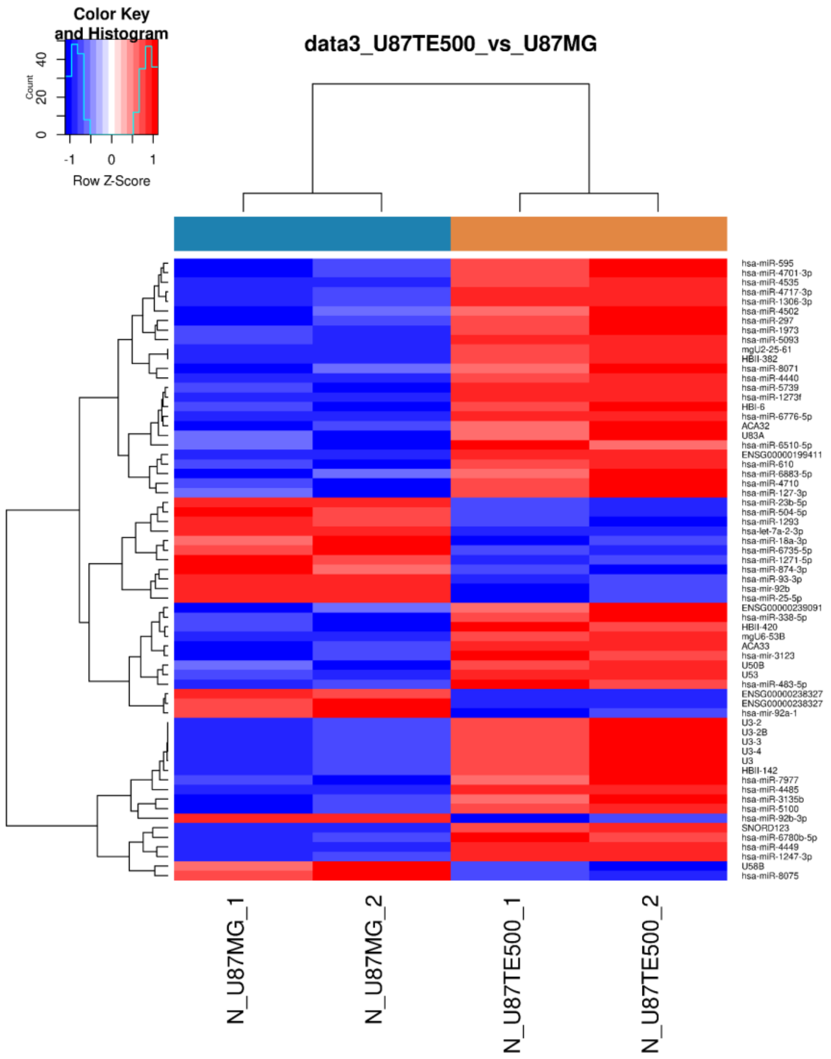

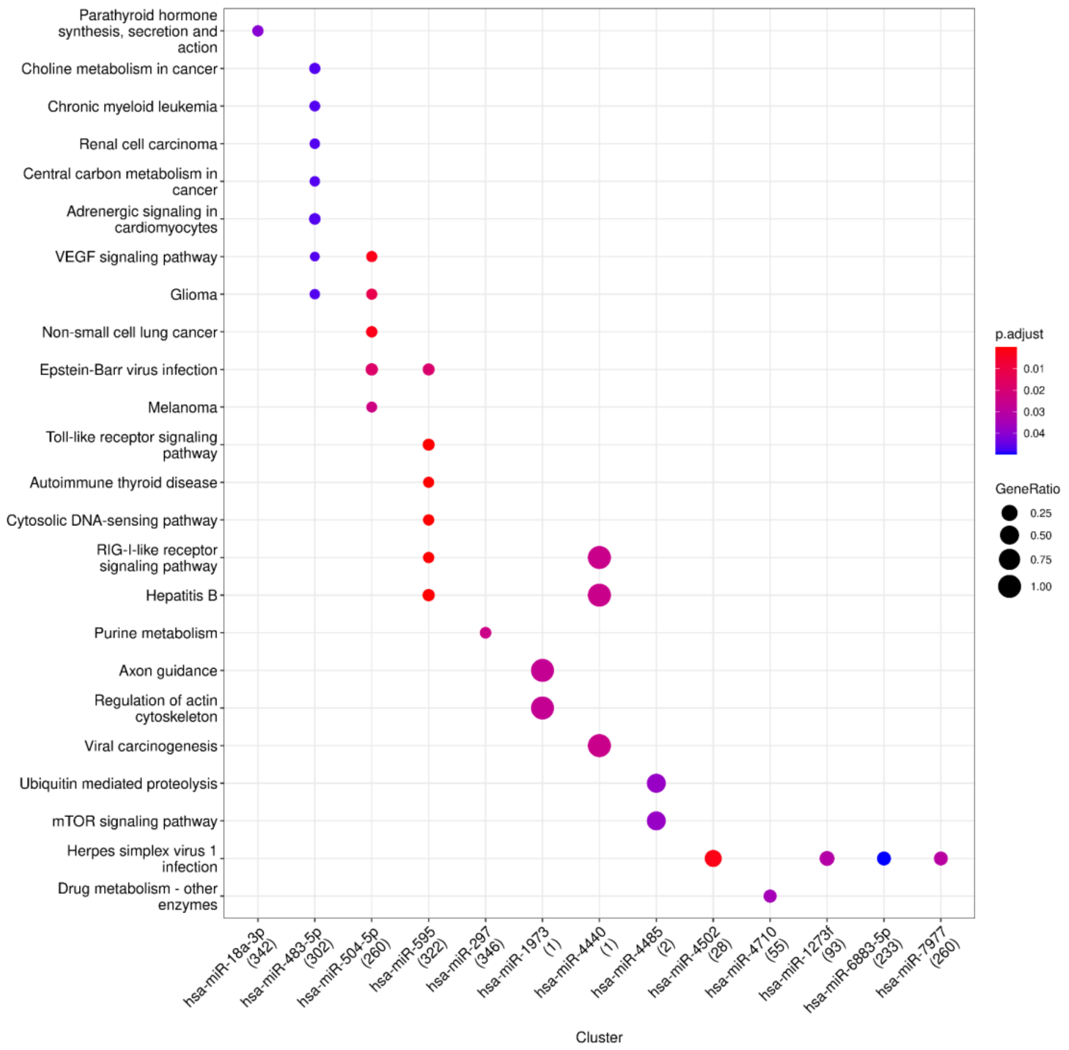

U87
TMZ vs. Evo/TMZ

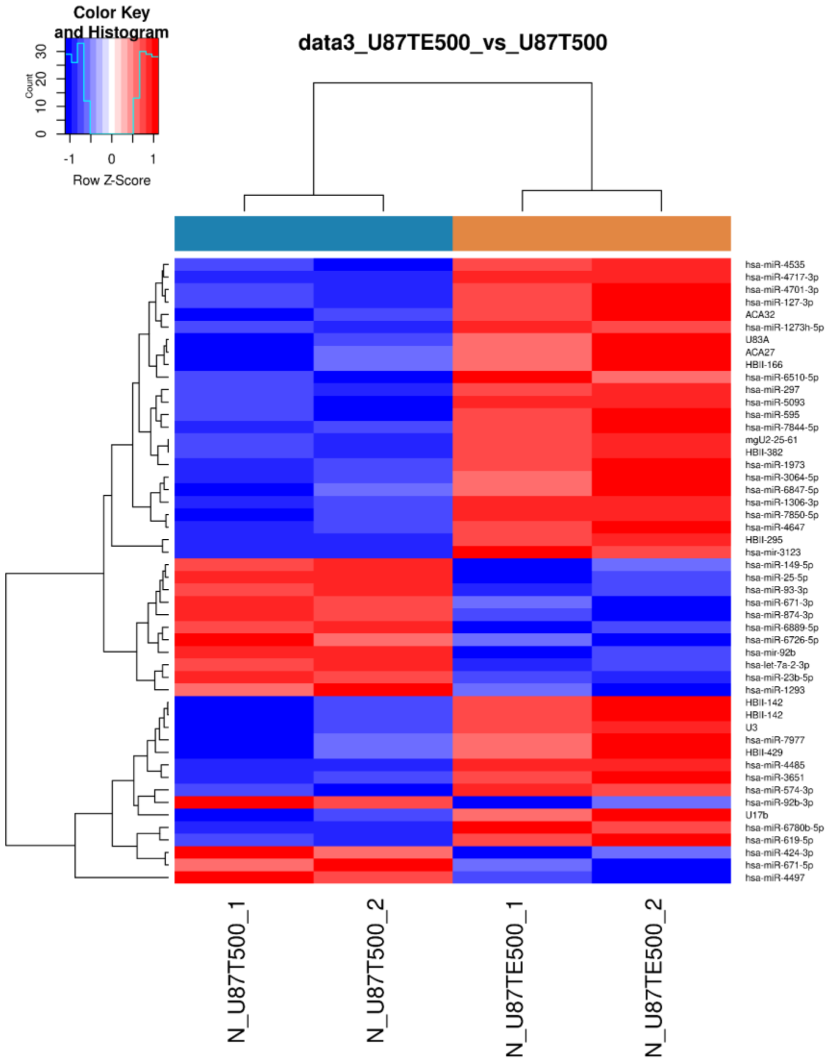

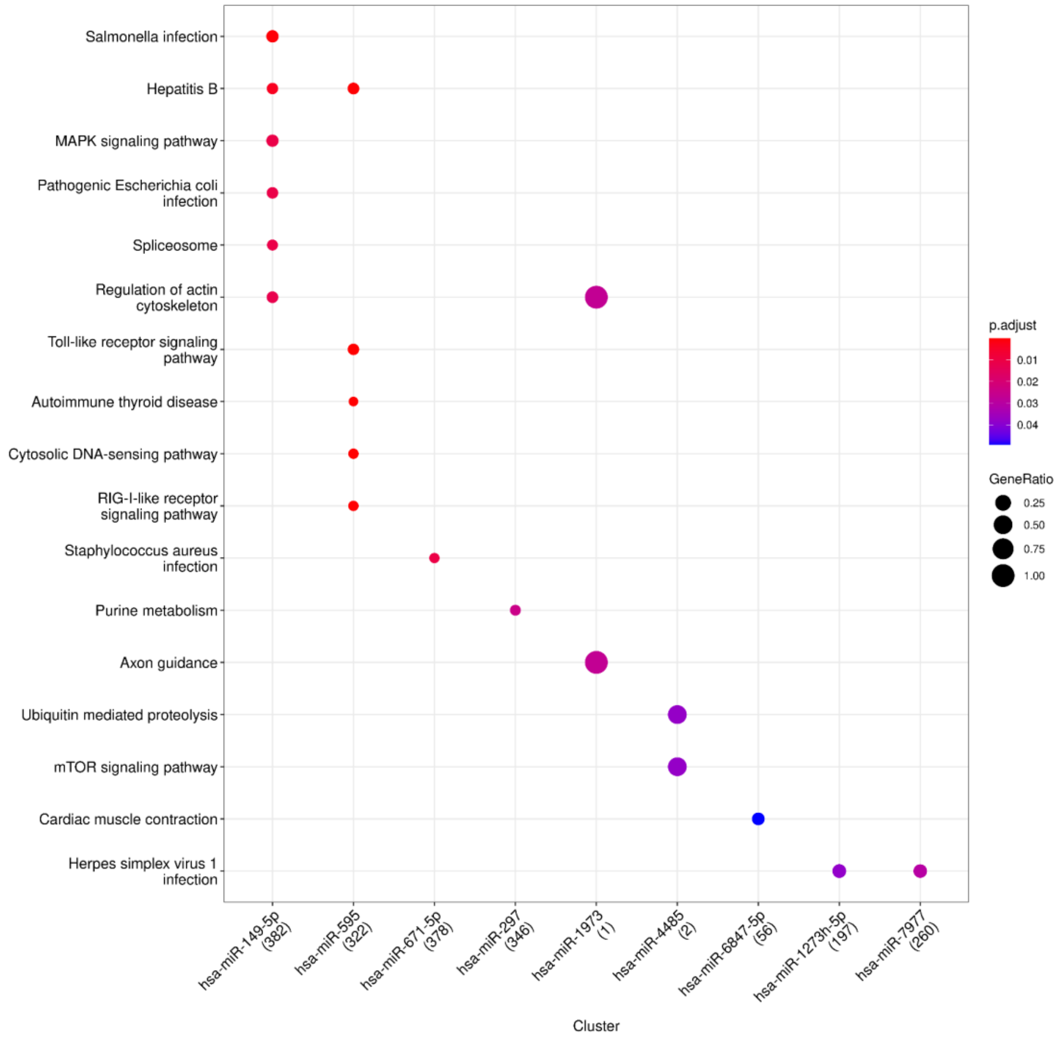

U373

U373
Control vs. TMZ

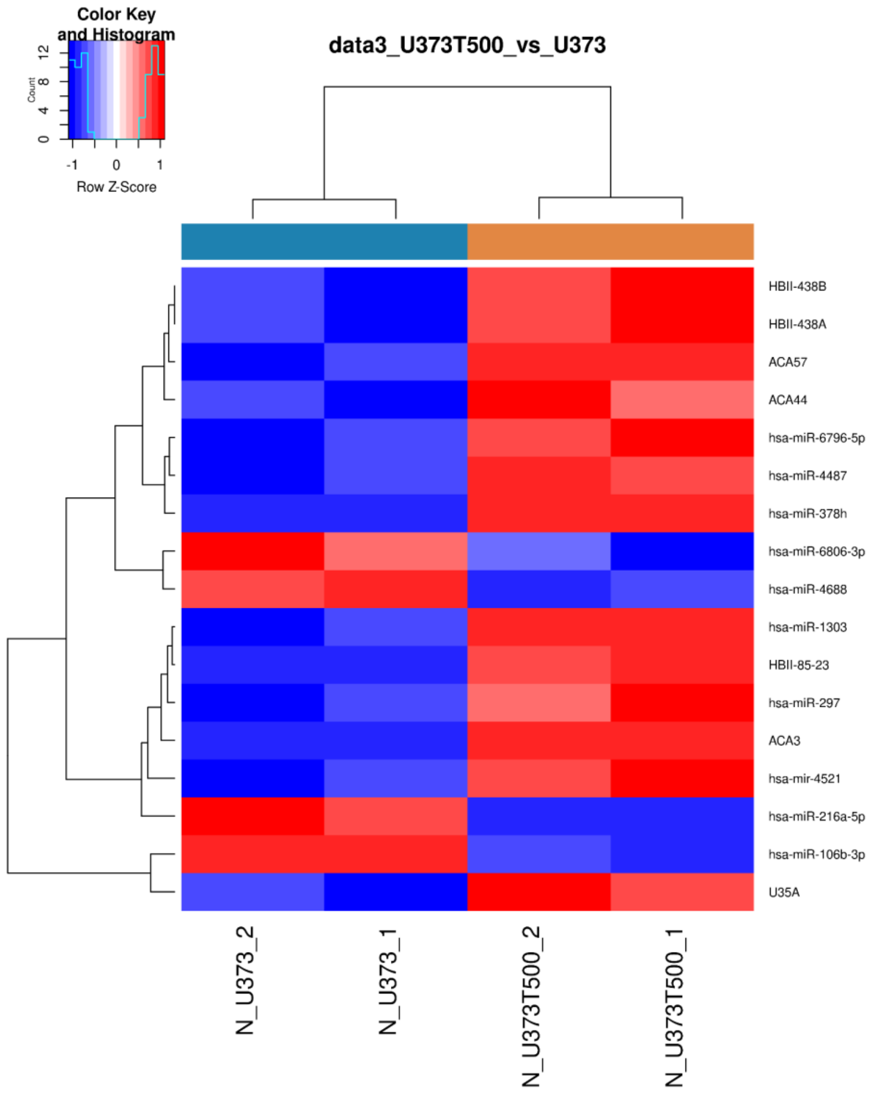

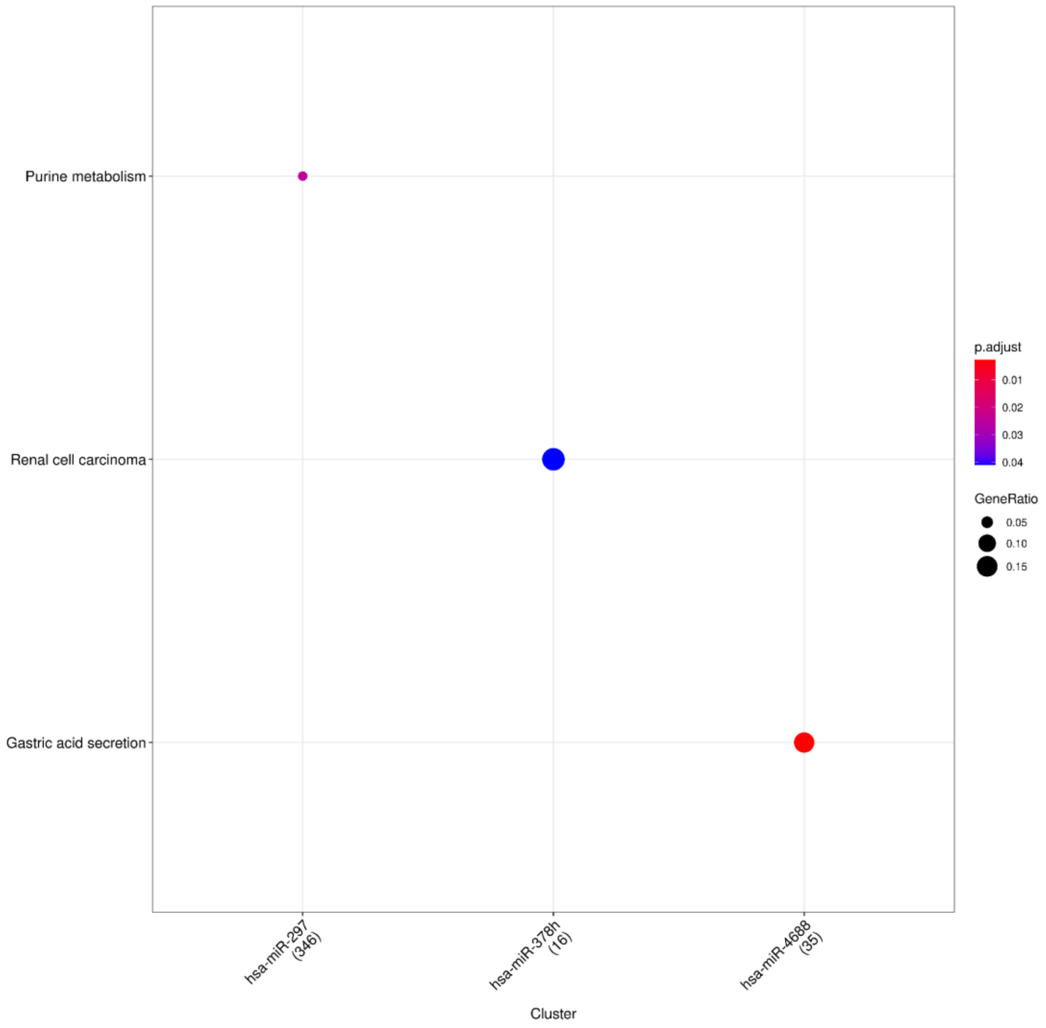

U373
Control vs. Evo

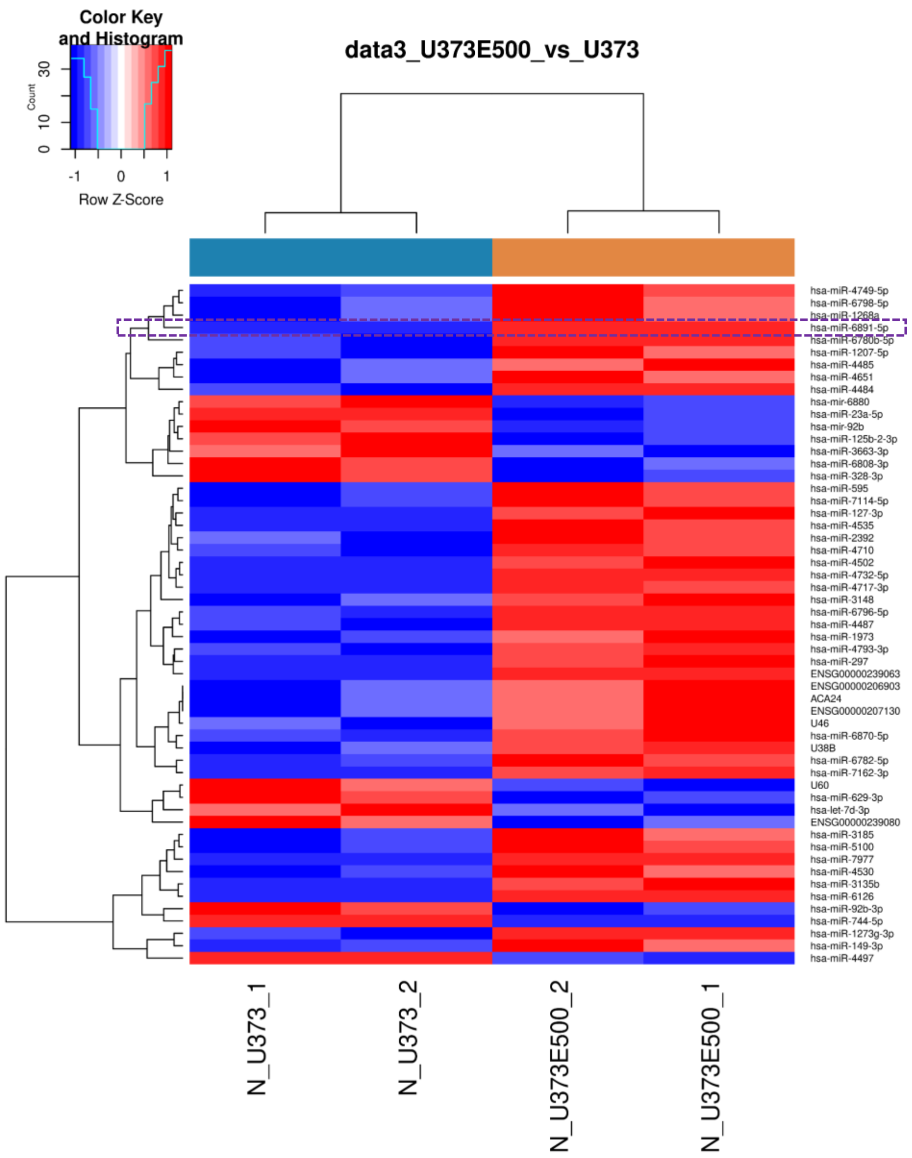

Result of Enrich KEGG pathway

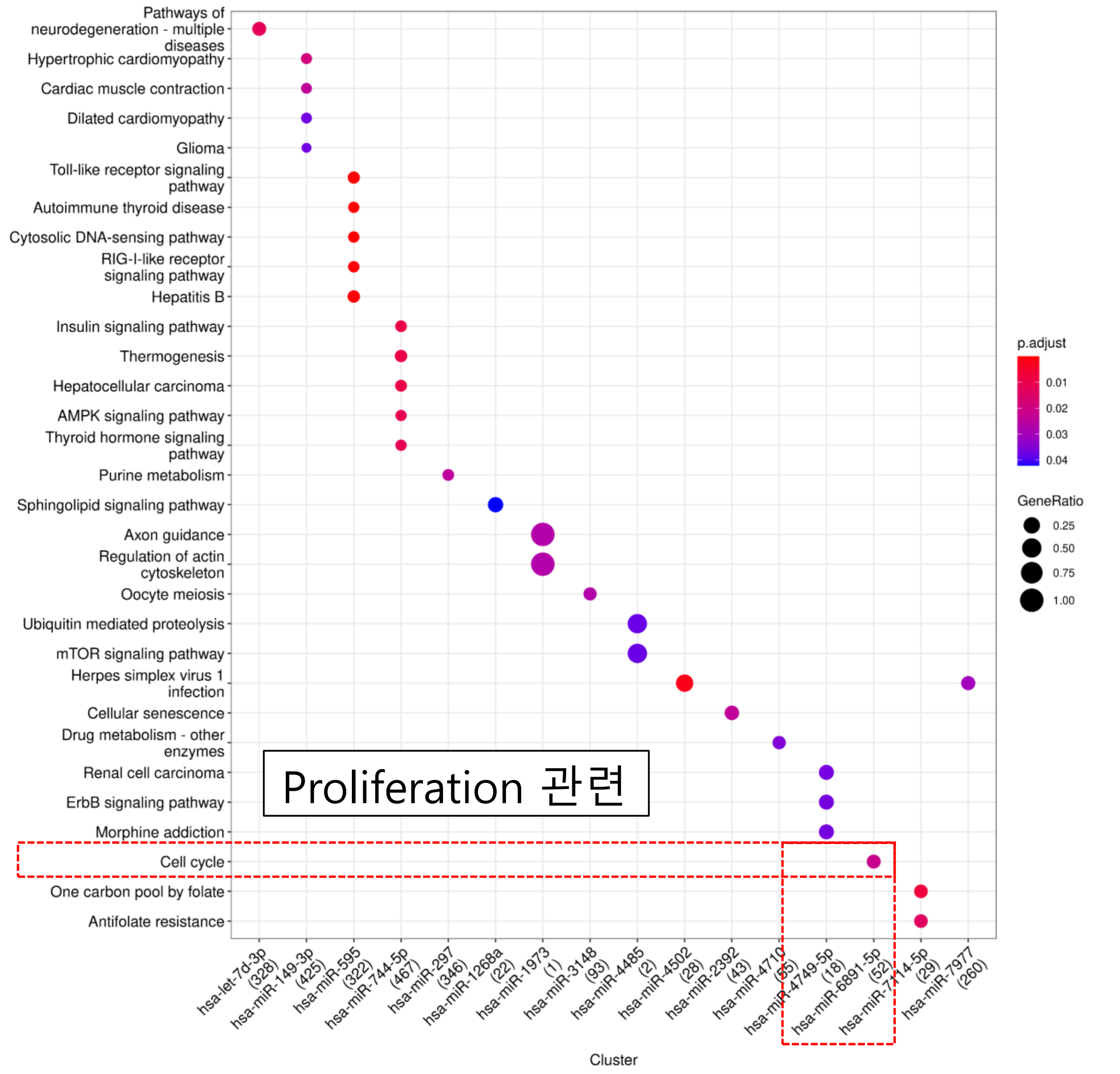

U373
Evo vs. TMZ

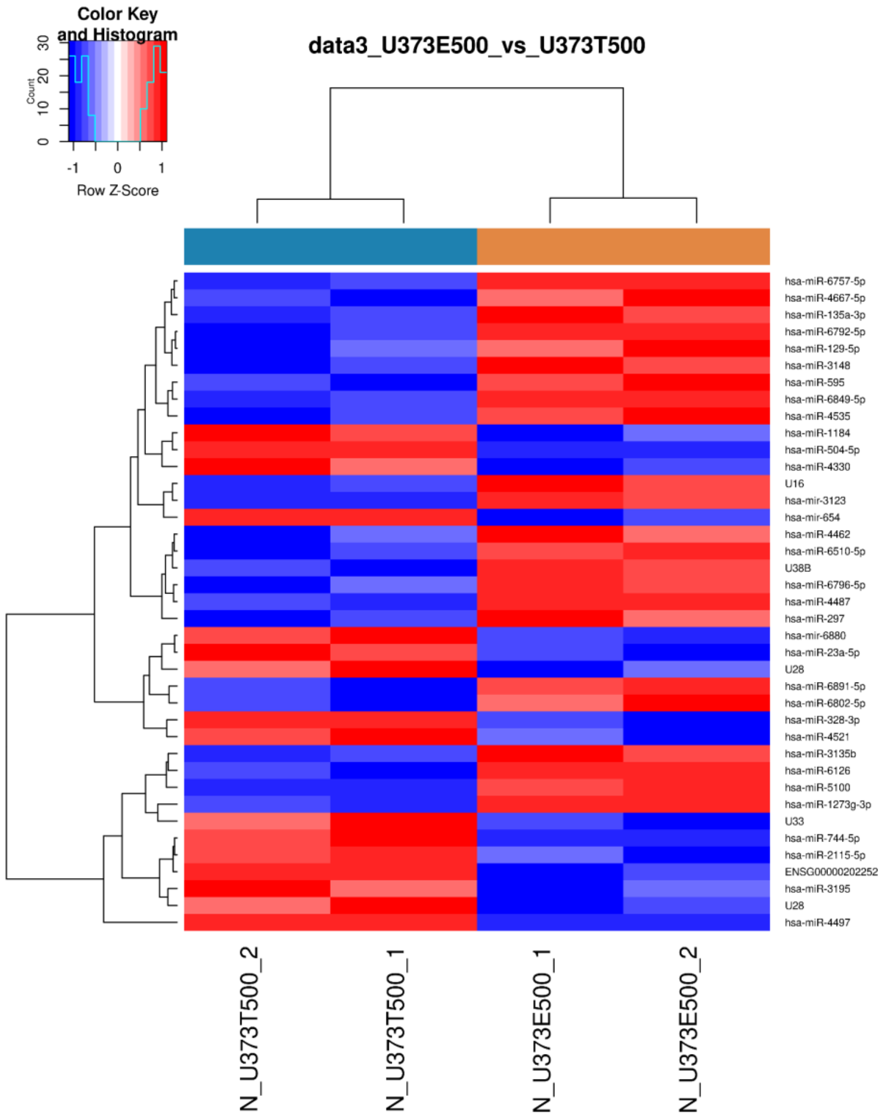

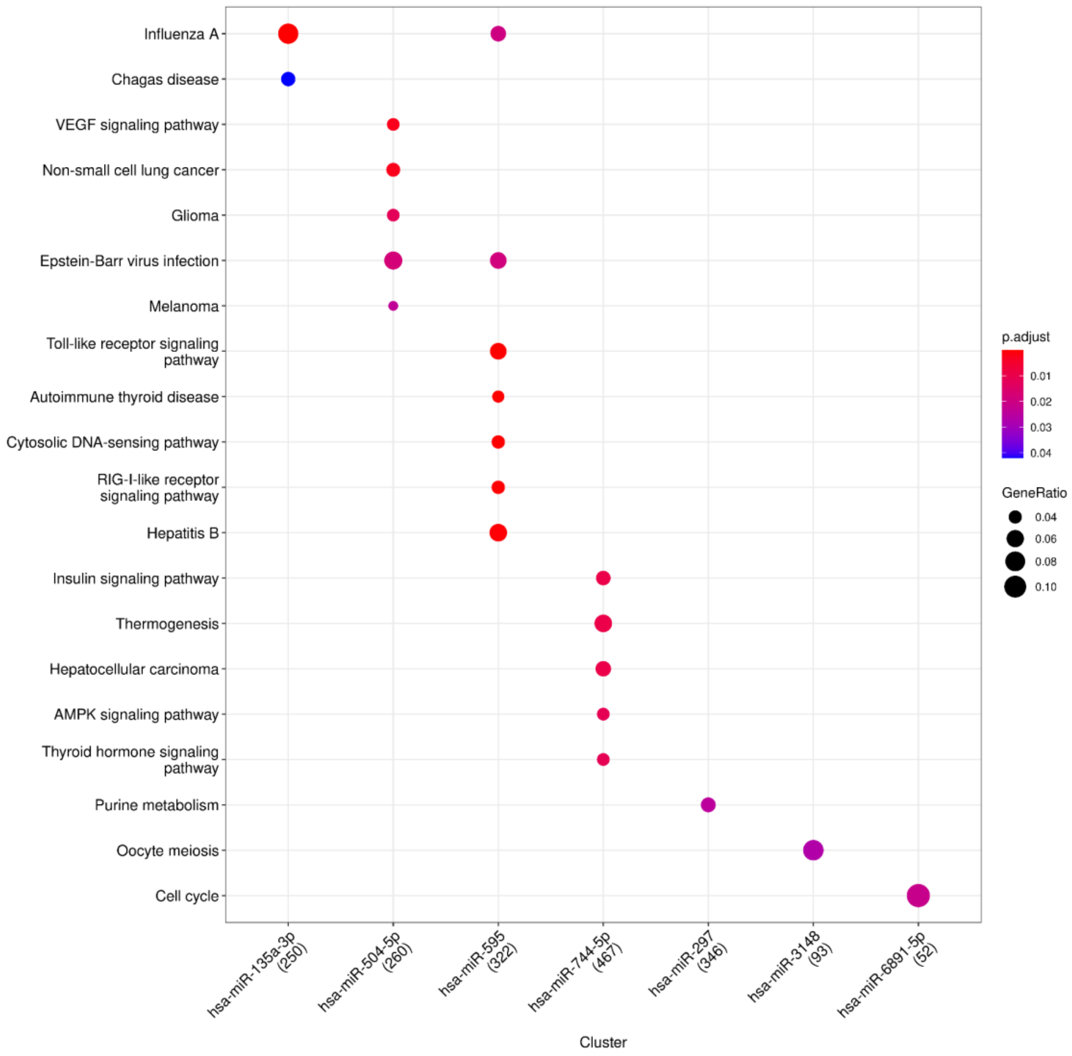

U373
Evo vs. Evo/TMZ

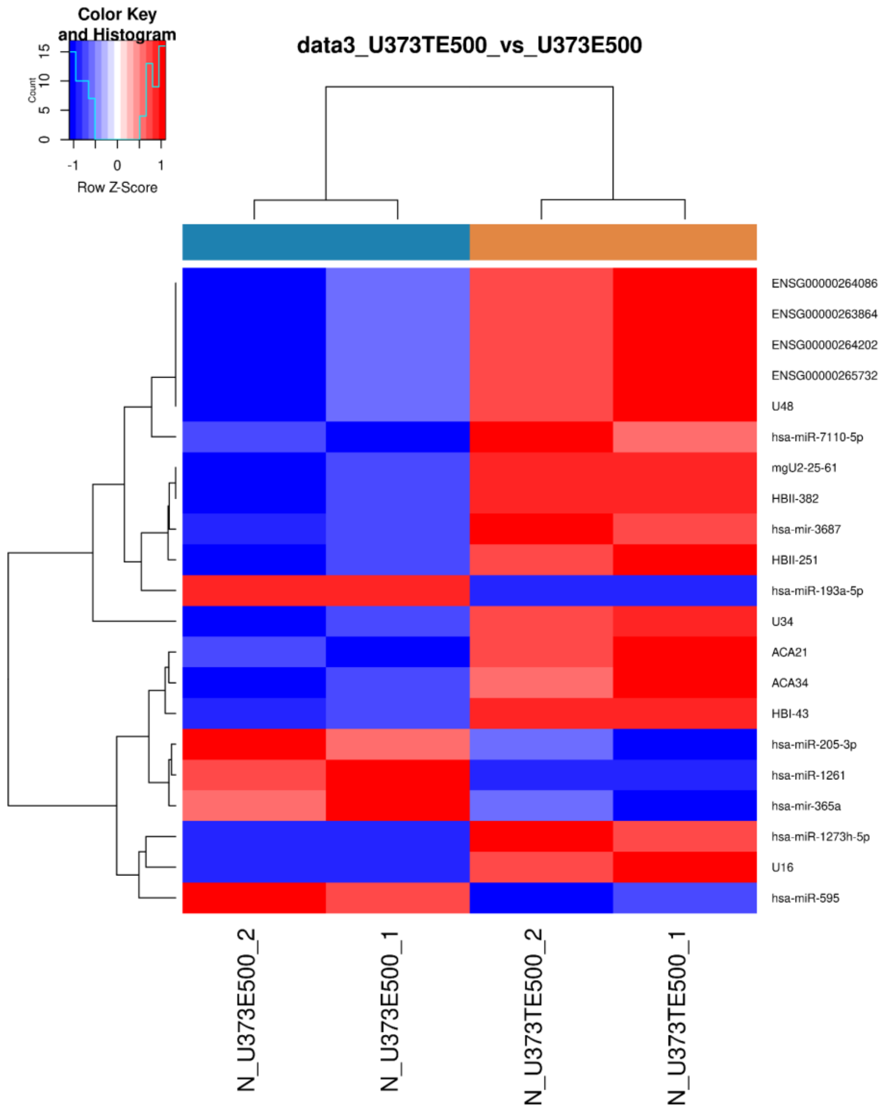

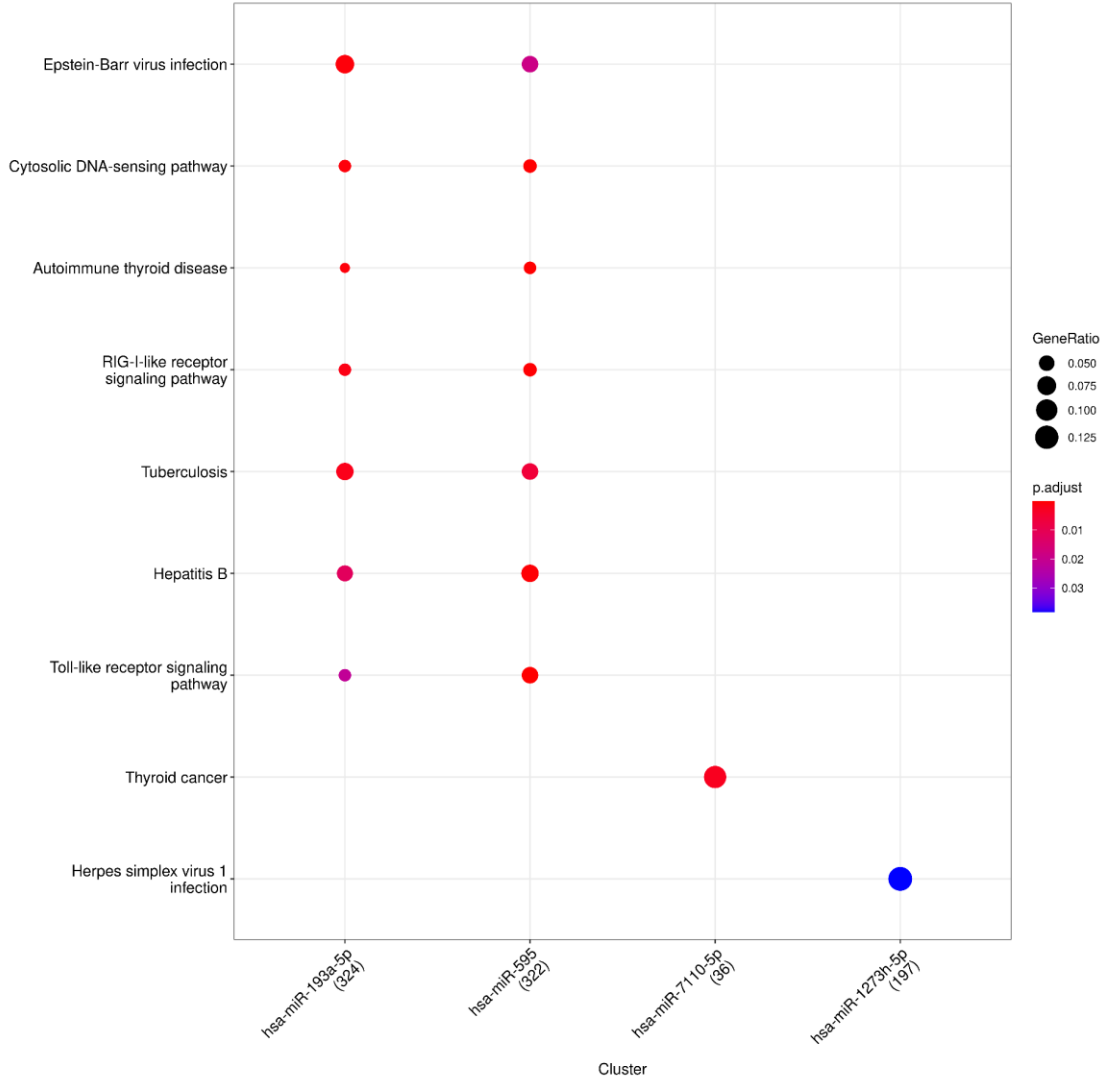

U373
Control vs. Evo/TMZ

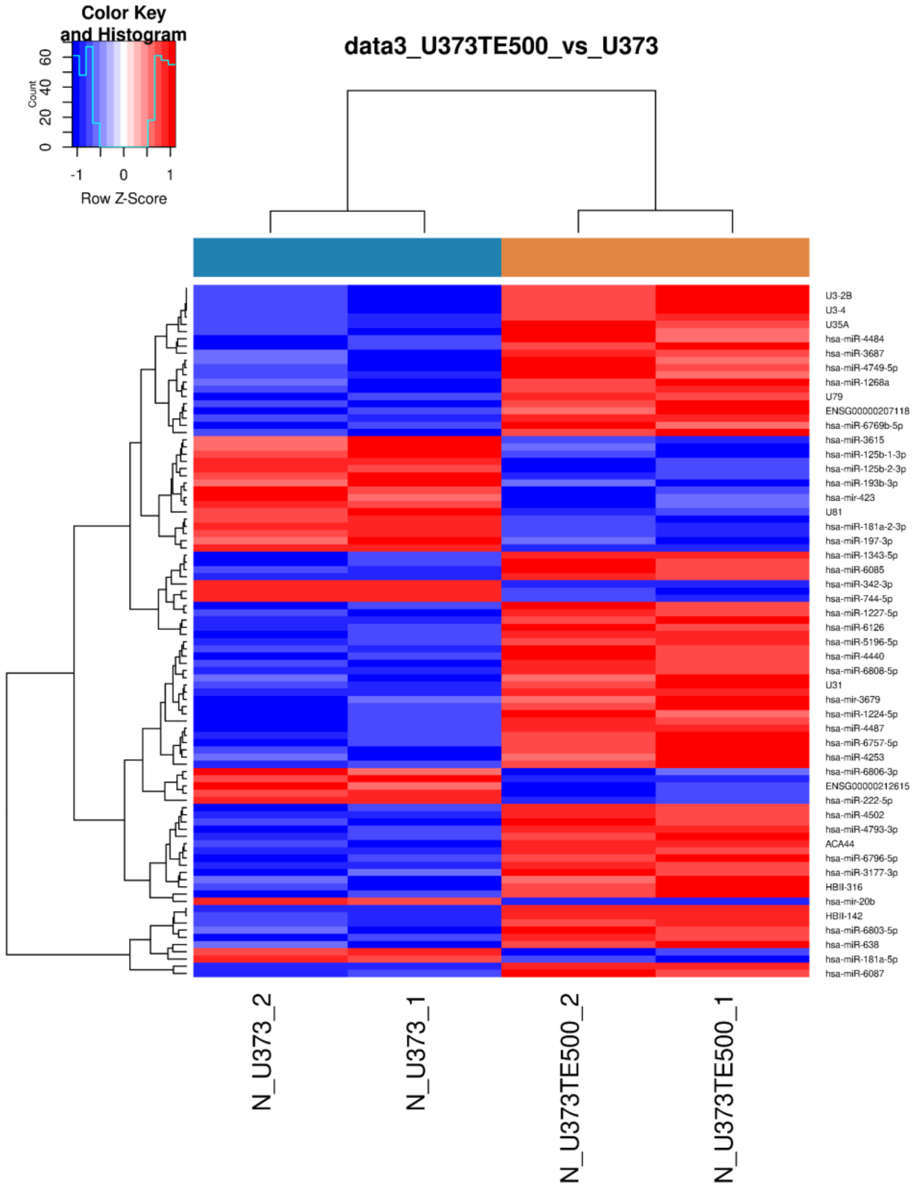

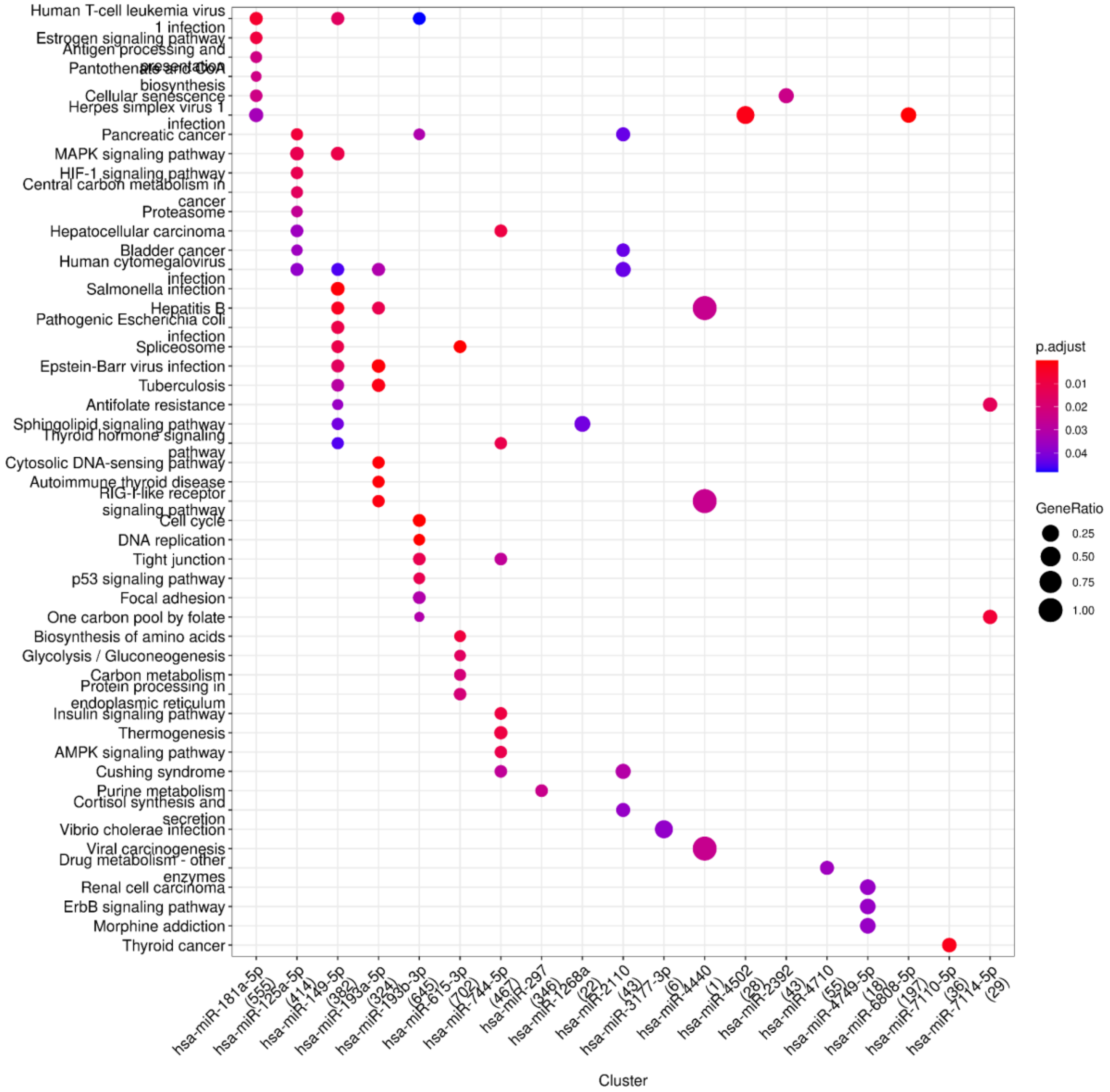

U373
TMZ vs. Evo/TMZ

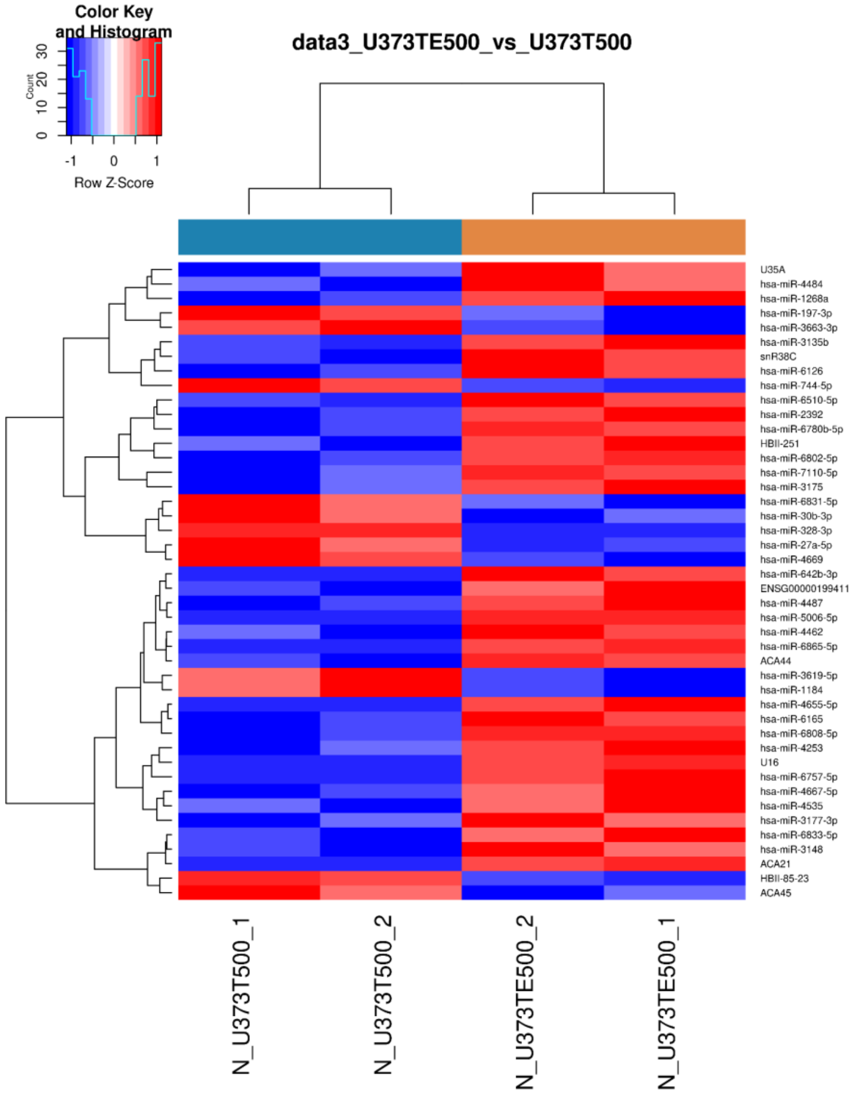

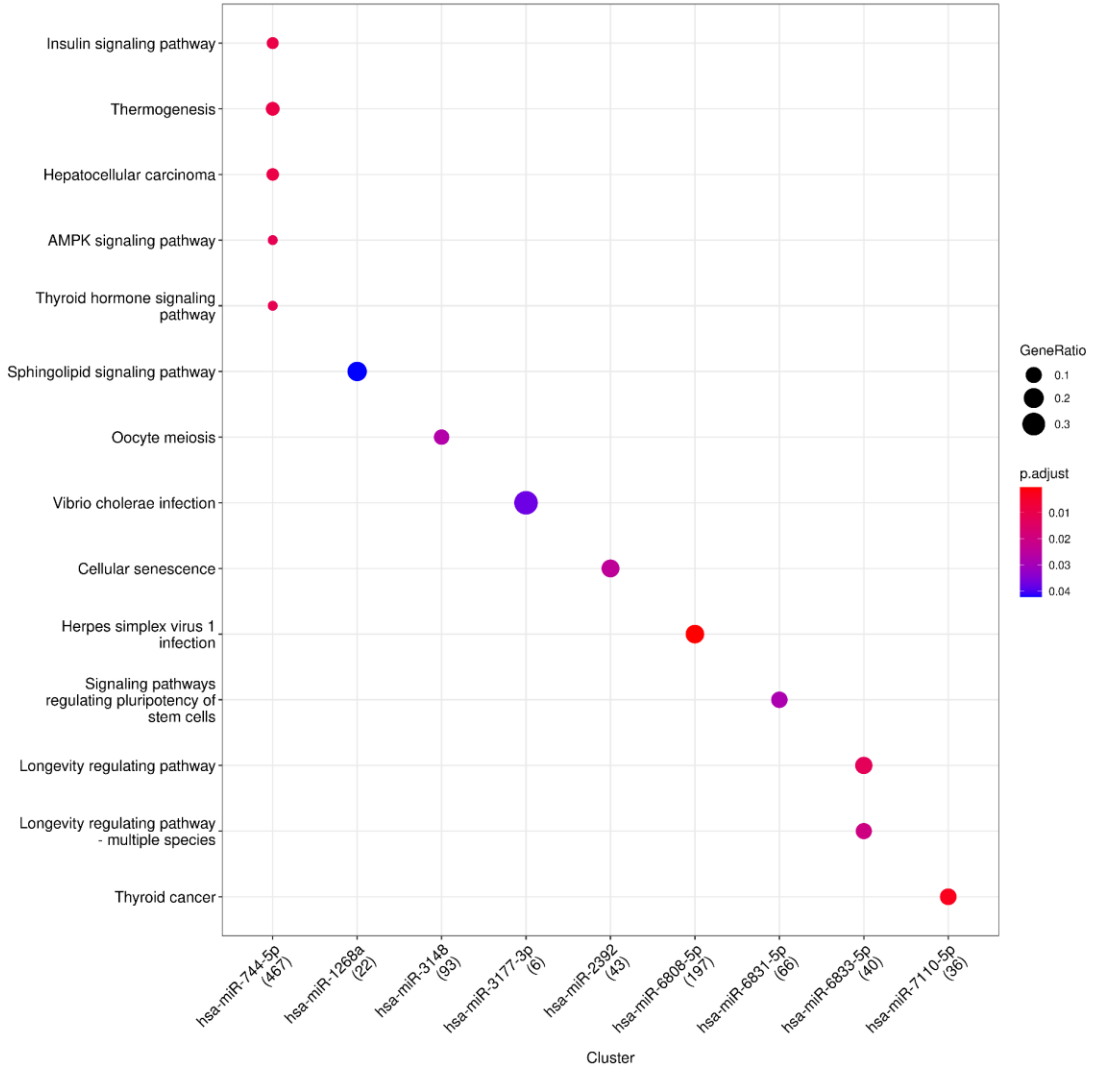

TCGA

Dipeptidyl peptidase-IV(DPP4)

Analysis of Survival

- ➢ DPP4_LGG

Median 3.46

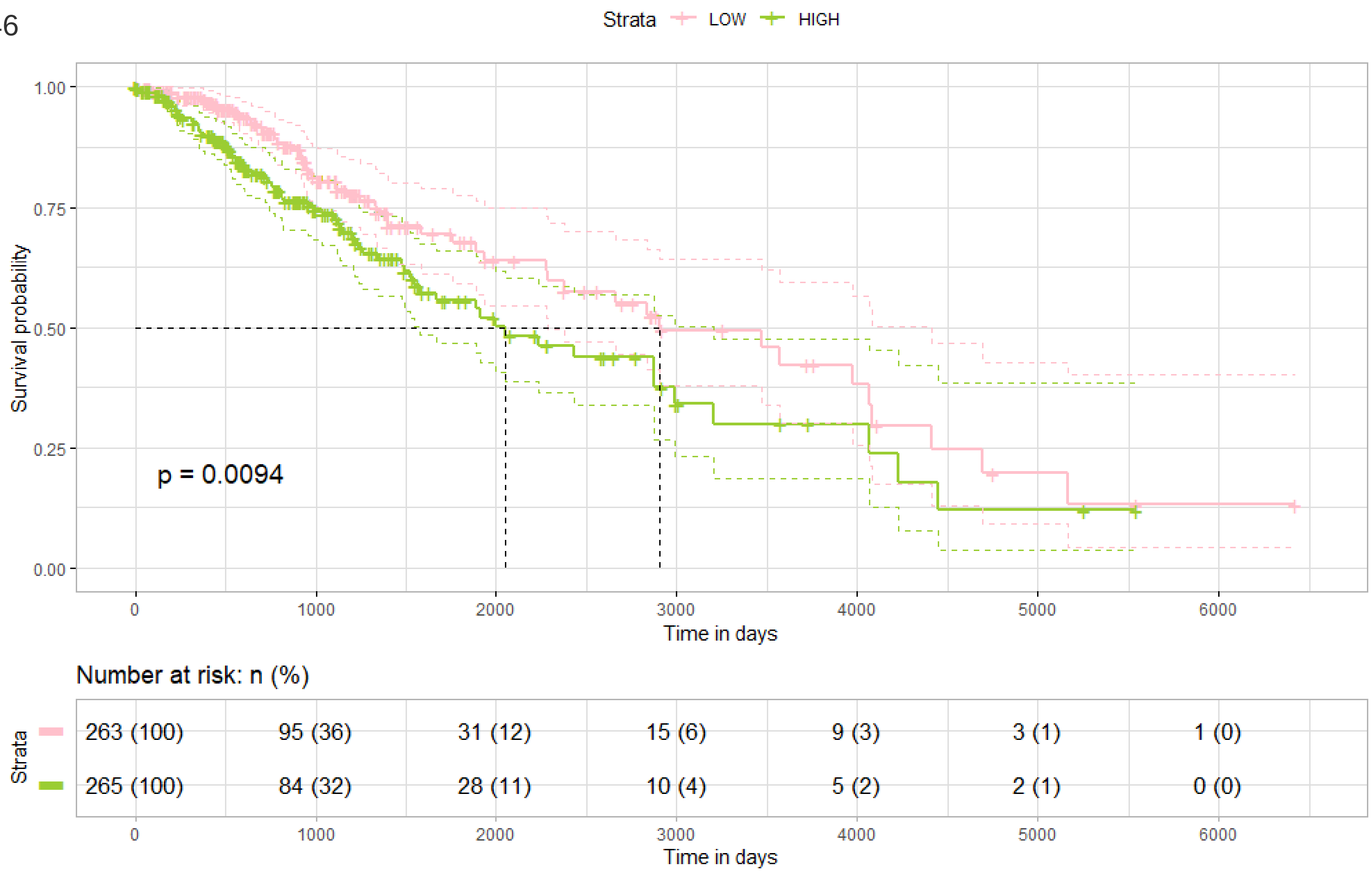

	records	n.max	n.start	events	*rmean	*se(rmean)	median	0.95LCL	0.95UCL
LOW=1	263	263	263	57	3101.863	230.8053	2907	2286	4412
HIGH=2	265	265	265	76	2528.416	232.3512	2052	1578	3200

Analysis of Survival

- DPP4_LGG

- Log rank test

```
         N Observed Expected (O-E)^2/E (O-E)^2/V
Def=1 263       57     71.8      3.07      6.74
Def=2 265       76     61.2      3.61      6.74

 Chisq= 6.7  on 1 degrees of freedom, p= 0.009
```

- Cox regression

```
  n= 528, number of events= 133

      coef exp(coef) se(coef)     z Pr(>|z|)
Def 0.4526    1.5725   0.1759 2.574   0.0101 *
---
Signif. codes:  0 ‘***’ 0.001 ‘**’ 0.01 ‘*’ 0.05 ‘.’ 0.1 ‘ ’ 1

    exp(coef) exp(-coef) lower .95 upper .95
Def     1.572     0.6359     1.114      2.22

Concordance= 0.583  (se = 0.025 )
Likelihood ratio test= 6.71  on 1 df,   p=0.01
Wald test            = 6.62  on 1 df,   p=0.01
Score (logrank) test = 6.74  on 1 df,   p=0.009
```

Analysis of Survival

- DPP4_GBM

Median 6.38

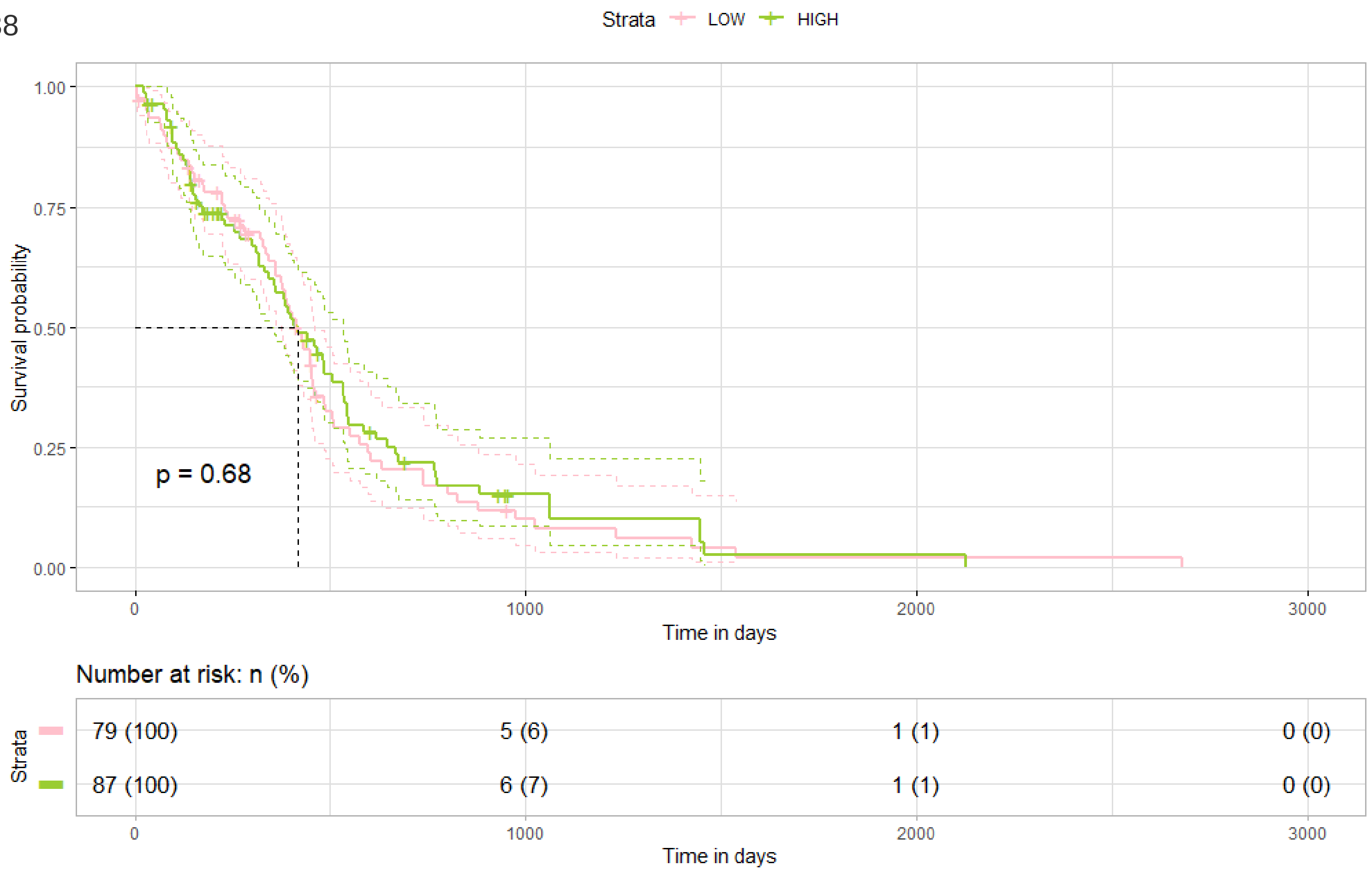

	records	n.max	n.start	events	*rmean	*se(rmean)	median	0.95LCL	0.95UCL
LOW =1	79	79	79	65	498.6761	54.28511	419	375	460
HIGH =2	87	87	87	68	529.7459	55.55769	419	342	535

Analysis of Survival

- DPP4_GBM

- Log rank test

```
       N Observed Expected (O-E)^2/E (O-E)^2/V
Def=1 79       65     62.7    0.0871     0.169
Def=2 87       68     70.3    0.0776     0.169

 Chisq= 0.2  on 1 degrees of freedom, p= 0.7
```

- Cox regression

```
        coef exp(coef) se(coef)      z Pr(>|z|)
Def -0.06904   0.93329  0.17466 -0.395    0.693

    exp(coef) exp(-coef) lower .95 upper .95
Def    0.9333      1.071    0.6627     1.314

Concordance= 0.504  (se = 0.026 )
Likelihood ratio test= 0.16  on 1 df,   p=0.7
Wald test            = 0.16  on 1 df,   p=0.7
Score (logrank) test = 0.16  on 1 df,   p=0.7
```
